# The rise and fall of memories: Temporal dynamics of visual working memory

**DOI:** 10.3758/s13421-025-01718-9

**Published:** 2025-05-06

**Authors:** Andre Sahakian, Surya Gayet, Chris L. E. Paffen, Stefan Van der Stigchel

**Affiliations:** Experimental Psychology, Helmholtz Institute, Utrecht University, Heidelberglaan 1, 3584 CS Utrecht, The Netherlands

**Keywords:** Visual working memory, Copying task, Encoding time, Retention time, Active vision

## Abstract

Visual working memory (VWM) is a cognitive system, which temporarily stores task-relevant visual information to enable interactions with the environment. In everyday VWM use, we typically decide how long we look to encode information, and how long we wait before acting on the memory. In contrast, VWM is typically studied in unnaturally rigid paradigms that keep presentation times and delays fixed. Here, we ask how visual memories build up over self-paced viewing times, and how they decay over self-paced delays, in a task that naturally engages VWM. We employed a copying task in which participants were tasked to recreate an “example” arrangement of items in an adjacent empty “workspace”. We tracked their unconstrained viewing and copying behavior at the level of individual items’ viewing times and the time to successful placements (i.e., delay). Our results show that performance monotonically increased for viewing times up to 1 s (per item), and plateaued afterwards. Interestingly, while views exceeding 1 s did not strongly improve performance for short (2-s) delays, views beyond 1-s did improve performance for longer delays. In contrast, this pattern was not observed in Experiments 2A and 2B, where viewing and delay times were experimentally manipulated (i.e., in more typical, rigid paradigms). These findings showcase the importance of considering aspects of naturalistic behavior, like decision-making, when studying VWM. We suggest that in everyday situations, short glances are sufficient for immediate use from VWM, but long views are required for effective delayed use.

**Supplementary Information:**

The online version contains supplementary material available at 10.3758/s13421-025-01718-9.

## Introduction

Visual working memory (VWM) is an essential cognitive function in our everyday behavior (Baddeley, 1986; Logie, 1995). It allows us to maintain visual information for a short time period in order to smoothly and efficiently interact with our surroundings (Heuer et al., 2020; Nobre & Stokes, 2019). Keeping visual information available is important, because relevant objects are often removed from sight temporarily, for instance due to shifts in gaze direction (i.e., looking at something else), or due to occlusion by other objects. VWM can store relevant visual information, so humans can plan actions and successfully execute them in a dynamic visual environment.

For example, to safely cross a multi-lane intersection, we rely heavily on our visual working memory. When we have looked in one direction, we need to remember what was there while we look in the other direction. Or when a car moves out of sight behind a parked bus, we need to keep the car in memory to safely cross the street. Even though we know a great deal about VWM from decades of research (see Brady et al., 2024 for a recent review), a relatively small portion of the literature has focused on the time course of visual memories: how they are built up over time (Bays et al., 2011; Bundesen, 1990; de Jong et al., 2023; Donkin et al., 2015), and how they decay over time (Donkin et al., 2015; Oberauer & Lewandowsky, 2008; Souza & Oberauer, 2015; Souza et al., 2016; Zhang & Luck, 2009). Thinking of the traffic example again, while we look at a vehicle, a memory representation presumably gradually builds up (i.e., comprising its size, location, velocity, etc.). When the vehicle disappears from sight, the quality of this memory representation decays over time, and new incoming information might interfere with the representation of the vehicle. Our current goal is to uncover how the duration of encoding and the time until utilization jointly determine how well information can be utilized in a naturalistic context. In doing so, we can learn what the most time-efficient course of action is: How long do we *need* to look at relevant visual information to enable successful actions? How long can we wait before the stored information has decayed so much that it cannot be utilized successfully anymore?

Our main goal naturally divides into three questions: (1) How are visual memory representations built up during visual inspection? (2) How do they decay while being retained in VWM? And (3) Does the time spent to build up representations influence the decay of these representations? Importantly, we want to investigate the three posed questions during naturalistic memory use. Note that we use the term "naturalistic" throughout the article in referral to two specific aspects of real-world VWM use that do not apply to typical VWM experiments. The first aspect is that the task is self-paced: participants can decide for themselves how long they look at relevant information, and how long they wait before using it. Secondly, the task does not force any one particular approach: participants have unrestricted access to task-relevant information, and can thus freely choose to memorize any number of items in any order (even multiple times) before acting.

Typically in visual working memory studies, answering the main question requires an experiment with a rigid task structure. Therefore, in the vast majority of VWM studies, both the presentation time of memoranda and the time until a report is prompted are fixed. To illustrate, when investigating whether memory performance is better for familiar items (an image of a car) than abstract items (a scrambled image of a car), both types of items should be presented for the same duration, and should be probed after the same delay period (Asp et al., 2021). The upside of this approach is that any variability in the outcome measure cannot be explained by (strategic) changes in encoding or retention time, so that any variation in performance can be explained by the experimental manipulation of interest. The downside of this approach, however, is that it does not reflect working memory use during real-world, self-paced behavior. Natural contexts allow for different encoding and retention strategies, which can be tuned to the task context or stimulus type. Thus, these temporally rigid paradigms offer little insight in the interplay of variable encoding times and retention times, which play a large role in everyday behavior. Still, there are some studies which have parametrically manipulated for how long information was presented (Brady et al., 2016; Bays et al., 2011; de Jong et al., 2023). These studies have revealed a typical pattern of memory performance: performance initially increases with longer presentation times, and then plateaus for even longer presentation times. Another downside of a fixed temporal structure is that we cannot be certain that observers actively encode the information during the entire presentation time. Observers might disengage before stimulus presentation ends (i.e., encoding time might be shorter than presentation time), which could explain the plateauing of performance. In a self-paced task design though, much like in natural behavior, the viewing time will likely reflect the actual encoding time more closely, as we assume that when observers are free to look at other relevant information they will do so when they are done encoding. There are also studies regarding retention times (or memory decay), which have shown that longer retention times (perhaps unsurprisingly) result in worse memory performance (Donkin et al., 2015; Oberauer & Lewandowsky, 2008; Rademaker et al., 2018; Schurgin et al., 2020; Souza & Oberauer, 2015; Souza et al., 2016; Zhang & Luck, 2009). Care must be taken, however, in assigning strictly negative effects to delay periods, as there is quite some work showing positive effects of delays (Brown et al., 2007; Souza & Oberauer, 2017). For example, it is proposed that due to short-term consolidation, with longer delays (or longer inter-item intervals during sequential presentation) recall performance is boosted (Jolicœur & Dell’Acqua, 1998; Ricker & Hardman, 2017; but see Mızrak & Oberauer, 2021). Although in the context of the current task where both viewing time and delays are self-paced, it is not evident when (during viewing or afterwards) consolidation would occur. Perhaps, during self-paced viewing one views a next item not just after encoding (as stated before), but after consolidating the item currently in view. All considered, to the best of our knowledge, no working memory study has directly investigated interactions between encoding times and delays. For example, one possible interaction might be that longer encoding times produce more robust representations (i.e., representations that decay slower). An interesting parallel might be found in the related field of long-term memory, in which the equivalent interaction *has* been studied extensively. There are studies reporting a slower rate of forgetting, with a higher degree of initial learning (Baddeley et al., 2019; Stamate et al., 2020; Yang et al., 2016). Yet, there are also studies reporting equal forgetting rates regardless of initial degree of learning (Allen et al., 2019; Rivera-Lares et al., 2022; Slamecka & McElree, 1983). Thus, the dynamics of long-term memory do not provide any clear-cut predictions for our study of the dynamics of working memory. Notably, there might be many reasons why the findings above appear to diverge (e.g., different memory contents, different methods), and elucidating those is beyond the scope of the current study, which centers on working memory. While long-term memory and working memory rely on vastly different storage mechanisms, studies investigating the dynamics of long-term memory typically also employ temporally rigid paradigms (albeit with much longer durations). To fill the gap of knowledge about the temporal development of working memories in everyday contexts, we employed a self-paced paradigm which allows for, and encourages a more natural use of VWM.

There have been a handful of studies, which investigated VWM use in a self-paced task design (Ballard et al., 1995; Böing et al., 2023; Draschkow et al., 2021; Fu & Gray, 2000; Hayhoe et al., 1998; Hoogerbrugge et al., 2023; Koevoet et al., 2023; Melnik et al., 2018; Morgan et al., 2009; Sahakian et al., 2023, 2024; Somai et al., 2020). Most of these studies employed some variation of a “copying task”. The goal for a participant in such a task is typically to recreate an arrangement of items from an example (which is somewhat reminiscent of assembling a jigsaw puzzle by looking at the cover). Participants pick up items from a separate item pool and position them within an initially empty workspace to copy the example arrangement. Importantly, participants are allowed to look at the example as often and as long as they want. Akin to typical VWM experiments, participants in this paradigm need to encode information in VWM, retain this information for some time and utilize it. The way in which this task engages VWM, however, is much more similar to daily VWM use: the visual environment is stable and accessible for inspection at any time. Therefore, participants do not need to memorize all information that will become relevant for imminent action, but can choose to memorize only part of it, and encode the rest later (contrast this to the typical VWM paradigm in which all information must be memorized within a predetermined short time frame, after which the information will not be available anymore) (Van der Stigchel, 2020).

Of particular importance to the present study, in the copying task, participants are also free to choose how long they encode information into VWM, and how long they wait before they apply this memorized information. Due to the self-paced nature of the task and the various strategies that are used, a natural spread in delays emerges. Everything considered, this task paradigm is ideally suited to answer the three questions we posed before. First: “How does the representation strength of information develop (increase) over time as it is in view?”, second: “How does the representation strength degrade over time as information is retained?”, and third: “How does the viewing time affect the degradation of representations over time?” Despite the fact that multiple studies employing the copying task paradigm have been conducted, these questions are yet to be addressed. Regarding previous copying task studies of ours in particular (Sahakian et al., 2023, 2024), the experimental set-up did not allow monitoring viewing times for individual items. Therefore, questions about encoding time and decay times of individual items could not be addressed. We designed the current experiment to overcome these previous limitations: here we precisely tracked which individual items were viewed, for how long they were viewed, and whether successful utilization of the items ensued.

In our study, we had participants perform a computer task in which they recreated arrangements consisting of eight items in a 4 $\times$ 4 grid. In order to track *where* (more specifically: at which item) participants were looking, we limited their view of the to-be-copied example grid: they could only see a small (item-sized) area around the mouse cursor.

Previous work showed that using such a cursor-directed aperture in copying task paradigms (Sahakian et al., 2023, 2024), produced very similar viewing behavior to other studies which employed eye-tracking (Ballard et al., 1995; Draschkow et al., 2021; Somai et al., 2020). By tracking, for each to-be-copied item, how long it was viewed, and whether or not that item was subsequently copied or not, we derived how the duration of viewing individual items predicted the ability to successfully apply the encoded information. Furthermore, by tracking the time between the end of a view and the (un)successful application of the viewed item, we derived how the delay predicted the ability to successfully apply the encoded information.

To briefly summarize our main results: First, we found that memory performance increased with longer viewing times, but only up to a certain duration (about 750 ms) after which further prolongation of the viewing times did not lead to better performance. Second, we found that memory performance monotonically decreased with longer delays. Lastly, and most interestingly, we found that even though longer views did not lead to substantially better performance on the short term (i.e., when it would be applied immediately), longer views did create more robust memories, which were successfully applied after long delays.

**Fig. 1:**
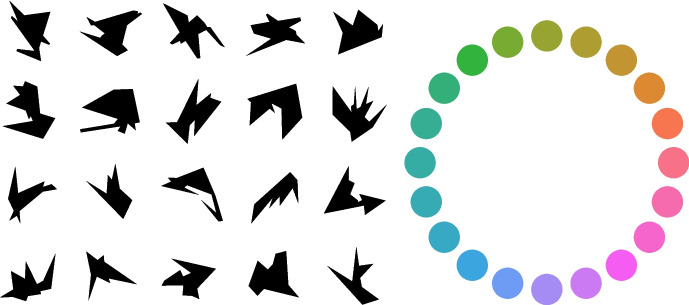
The 20 shapes and 20 colors that were combined to create the stimuli in the experiment. Given 20 shapes and 20 colors, we could create 400 unique stimuli. For each trial, a random selection (*without* replacement) of four shapes and four colors was used to create 16 unique stimuli (with the restriction that any two colors were at least three hues apart). From this pool of 16 stimuli, eight were randomly selected (*with* replacement), and randomly positioned in the *Model* grid for each trial

An open question at this point was whether this pattern of results would generalize to typical VWM paradigms. More specifically: is it a fundamental property of VWM that longer encoding produces more robust memories? If so, that would mean that an important property of VWM has been – to the best of our knowledge – overlooked in the current literature. We reasoned that if the interaction (i.e., slower decay of memories with longer views) is inherent to VWM, we should observe this interaction in any VWM paradigm, and definitely in the most conventional VWM paradigms. Therefore, to answer the posed question, we deliberately chose to stray from our initial design and use a more prototypical VWM paradigm.

Results from two additional experiments employing typical VWM paradigms (i.e., with a forced report after fixed viewing times and delays), suggested that it does not; longer views did not create more robust memories in typical VWM experiments with forced choice responses and fixed viewing times. In the General discussion, we suggest that the influence of viewing time on decay (as observed in the copying task alone) is *not* a fundamental property of VWM, but might be specific to unrestricted tasks, where participants can choose to withhold their response when certainty about a memory item drops below a critical threshold. This study provides the first steps to a comprehensive understanding of visual working memory use in natural interactions with the environment. Particularly, we argue that considering the self-paced nature of our interactions is essential to capture the temporal dynamics of naturalistic working memory use.

## Experiment 1: A naturalistic VWM task

### Methods

The methods are very similar, and in some aspects identical to previously published studies from our lab (Sahakian et al., 2023, 2024). We report the full methods here, but note that parts in this section may be paraphrased or copied from the methods sections of those articles.

#### Participants

Data of 49 participants were collected. In some trials of some participants, data were not recorded properly. After filtering out these trials, we selected the participants which had 25 or more properly recorded trials (of the 30 trials they performed). We included the data of 46 participants (35 females and ten males) in the formal analyses. The mean age of the included participants was 21.3 (SD = 1.59). The experiment was approved by the *Ethics Committee of the Faculty of Social and Behavioural Sciences of Utrecht University*. The approval is filed under numbers 23-1027 and 23-0605. All participants gave informed consent before beginning the task. They either received course credit or participated voluntarily without monetary compensation.

#### Apparatus and stimuli

The experiment was scripted using the JavaScript libraries jsPsych version 7.3.0 (de Leeuw, 2015) and Fabric.js version 5.2.4 (fabricjs.com). We used Gorilla to build and run the experiment (Anwyl-Irvine et al., 2020). Despite using tools primarily meant for online experiments, we ran this experiment in the lab. Data was collected in two identical rooms. Both rooms had a Windows PC connected to a Dell monitor (model: P2419H; screen size: 24 inch; resolution: $1920 \times 1080$ pixels; refresh rate: 60 Hz). The task was run on either the Google Chrome (version 108) or Microsoft Edge (version 109) browser. Participants used a computer mouse to complete the task.

**Fig. 2:**
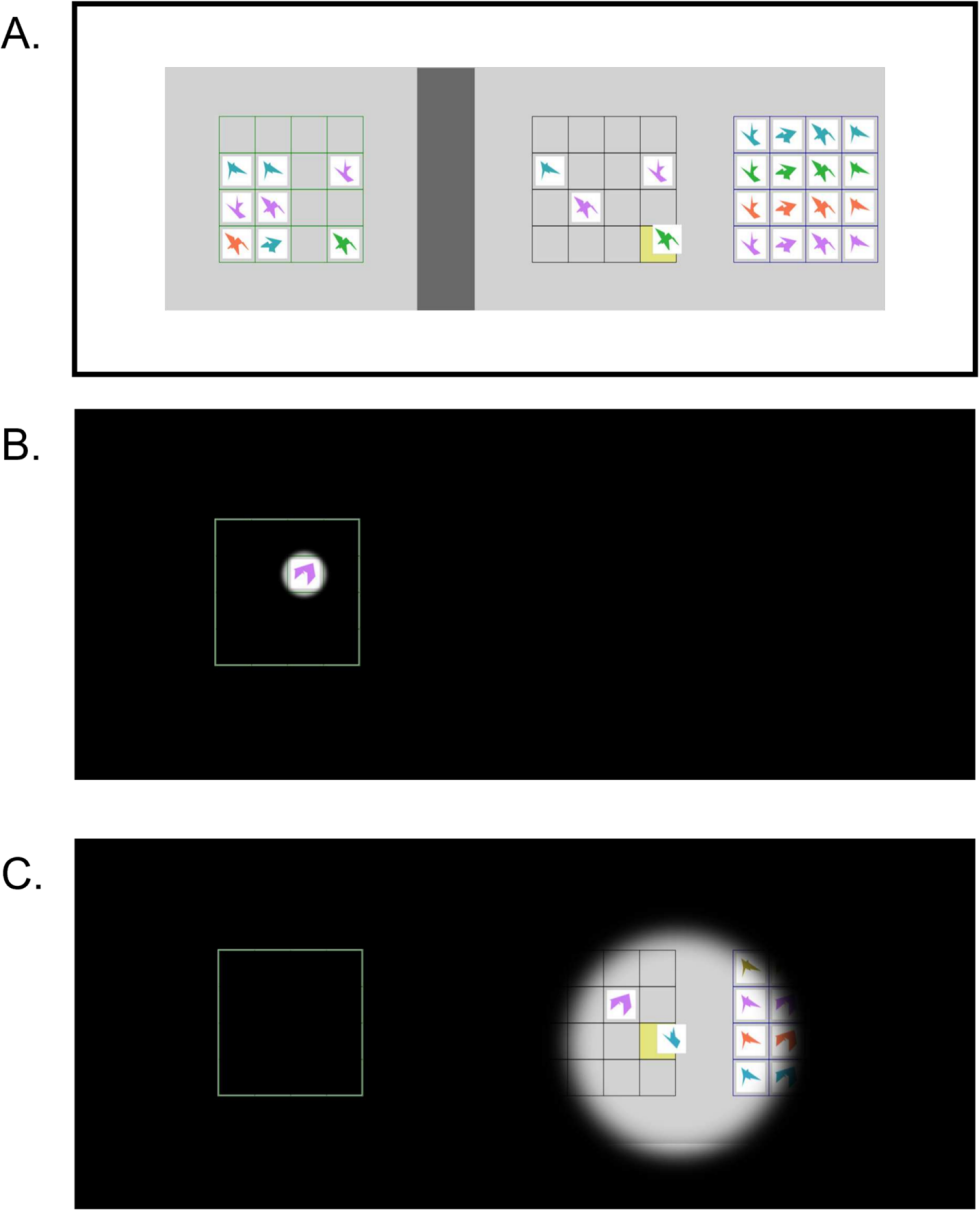
Overview of a trial in Experiment 1. **A** Overview of the task display. There were three relevant 4 $\times$ 4 grids. From left to right they were the Model-, Workspace-, and Resource grid. The Model needed to be recreated in the Workspace using the items from the Resource. The items were picked up and dragged to the correct location in the Model grid using the computer mouse. **B** The task view during experimental trials. A black overlay covered the entire task display. Only a circular area, always centered on the mouse cursor, was made visible. While the aperture was in the Model area (i.e., left of the dark gray bar) the aperture was just large enough to see one item. **C** The task view during experimental trials, but when the cursor was in the Workspace and Resource area (i.e., right of the dark gray bar). Here, the aperture was much larger: it provided a complete view of the Workspace grid. See https://osf.io/5pj8b for a demonstration video

The 20 shapes of the items were adapted from stimuli used by Arnoult (1956). The 20 colors were selected from the HSLuv color space (www.hsluv.org). Specifically, we selected 20 perceptually equidistant hues (starting at a hue value of 3.59 degrees, and adding 17.95 degrees to get the next hue value) on the color wheel, with the saturation set to 89.1% and luminance to 64.35% (see Fig. 1).

In order to track what part of the experimental display participants were looking at in this online study, we implemented a cursor-directed aperture in our task (Anwyl-Irvine et al., 2021). Effectively, this meant that a black overlay covered the view of the display, and only a circular area surrounding the location of the cursor was made transparent (see Fig. 2B and C; or see https://osf.io/5pj8b for a demonstration video).

The aperture was small (the size of an item) in the Model area, and was large (the size of a 4 $\times$ 4 grid) in the Workspace and Resource area. Specifically, the radius of aperture was set to respectively 3% or 15% of the width of the light gray experiment rectangle. The edge of the circular aperture was blurred by applying a Gaussian filter with a standard deviation equal to 10% of the radius of the aperture. The experience of the participant was similar to looking around in a darkened environment using a focal flashlight. The location of the aperture was recorded 60 times per second. To provide a cue as to where the Model grid was, a green outline of the Model was always visible. Without the outline it was unnecessarily hard for participants to determine in which cell an item was, as they had no overview of the entire Model grid.

#### Procedure

After giving informed consent, reading the instructions and completing two practice trials (one with a complete view of the task, and one with a black overlay and a cursor-directed aperture), participants started the main task. The goal in every trial was to recreate an arrangement of eight items in a four-by-four grid, called the Model grid. Participants did this by dragging and dropping items from the Resource grid onto the Workspace grid (see Fig. 2). When an item was placed in its correct location, it would be locked in place. When an items was placed incorrectly, however, the item would fly back to its original place in the Resource grid. Once the eight items were correctly placed in the Workspace, the trial was completed and the participant could move on to the next trial. There were 30 experimental trials. For each trial, a random sample of four different shapes and a random sample of four different colors (with the restriction that the distance between any two colors was at least 3) was used to generate 16 unique items (see Fig. 1). These 16 items comprised the Resource grid. On each trial, a new Model grid was created by selecting eight items (with replacement) from the Resource grid, and placing them in eight different locations. Selecting items with replacement entailed that the same item might have occurred more than once in a Model grid.

#### Measures

The two main measures were the viewing time and delay of individual items. To facilitate interpretation, we split the task execution into two alternating phases: 1) *Viewing sessions*, the phase in which the aperture was in the Model area and items were inspected; and 2) *Building sessions*, the phase in which the aperture was in the Workspace and Resources areas and items were dragged to the Workspace. To finish a trial, a participant would typically cycle through multiple repetitions of such sessions. We defined the viewing time of an item to be the time the cursor (i.e., the center point of the circular aperture) was inside the cell that contained the item. This would be the time that an item was visible to the participant.^1^ As the same item may have been inspected multiple times (within a viewing session and across multiple viewing sessions) our final outcome measure for viewing time was the sum of all viewing times of the item (i.e., the cumulative viewing time). We ignored the views of an item after it was placed correctly in the Workspace.

We defined the delay of a viewed item (our second measure) as the time from the end of the item’s viewing until it was correctly placed. At the end of a building session, some items that had been inspected were not placed. For these items, the delay was measured from the end of the most recent view until the end of the building session. Note that we disregarded errors, focusing only on whether or not viewed items were eventually placed.

At the end of each building session, we listed the items that were (at some point) inspected *and* not yet placed at the start of this building session. Some of these inspected items would be correctly placed at the end of this building session, and some would not be placed. For each inspected item (correctly placed or not) we would then determine its view time and delay as described above. Note that this procedure will result in some items appearing multiple times in the list.

**Fig. 3:**
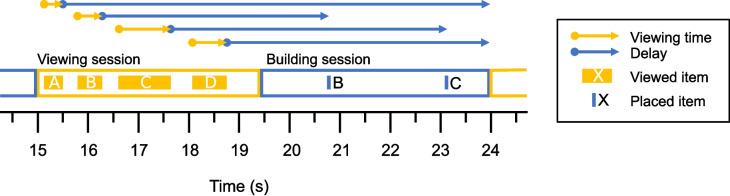
Schematic overview of the temporal measures in Experiment 1. This overview shows the events during a trial in a copying task on a time line, and matches the timings of events shown in Table 1. The *yellow outlined bar* represents a "viewing session": the time during which the aperture hovered over the Model area. During a viewing session, individual items are viewed. The *filled yellow bars* represent the time an individual item is viewed. The *yellow arrows* above the bars again represent the duration each item was viewed for. The *blue outlined bar* represents a "building session": the time during which the aperture hovered over the Workspace and Resource area. The *vertical blue lines* in the building session indicate that an item was (correctly) placed. The *blue arrows* above indicate the duration of the delay. Note that the delay of an item always starts immediately when the viewing of that item ends. The delay either ends when that item is placed, or at the end of the building session

To illustrate the procedure, we provide an example with (arbitrarily chosen) data (see Fig. 3). Let us say a trials starts at time point *t*=0.0 s. Some time into the trial, a viewing session starts at time point *t*=15.0 s, first item A is inspected for 0.3 s (*t* = 15.2 s - *t* = 15.5 s), then item B for 0.5 s (*t* = 15.8 s - *t* = 16.3 s), C for 1.2 s (*t* = 16.6 s - *t* = 17.8 s), and finally item D is inspected for 0.6 s (*t* = 18.1 s - *t* = 18.7 s). In the building session that follows, item B is placed 4.5 s after the end of item B’s view ended; then item C is placed after 5.3 s after the end of item C’s view ended; then this building session ends at *t* = 24.0 as the aperture enters the Model area again, without item A and D being placed. The list will be extended by these items with the associated viewing and delay times (see Table 1).

#### Analysis

To predict whether an item was placed or not, based on the viewing time and delay, we conducted a Bayesian generalized linear mixed model (GLMM) analysis, using the brms package in R Statistical Software (Bürkner, 2017; R Core Team, 2024). Using a GLMM is preferable as it allows us to consider each data point (i.e., a row from the table as illustrated in Table 1). The alternative would be to pool data into arbitrarily sized bins, which might inadvertently introduce biases. We compared a full model, with fixed effects ’Viewing time’ and ’Delay’ as well as their interaction against the same model but without the effect of interest. Note that we included the ’Trial number’, ’Viewing session’ and ’Number of item views’ within a trial as fixed effects as we assumed they would have predictive value, and to ensure the effects of interest were not driven by these factors. We also added a random intercept and random slopes for the effects of interest for each participant (Oberauer, 2022). In Wilkinson notation, the full model was specified as follows: *Placed* $\sim$ Viewing time * Delay + n-th Trial + n-th Viewing session + n Item views + ( 1 + Viewing time * Delay | Participant). The model comparisons yielded Bayes factors indicating how much more likely it is to observe the data given one model versus the other. We obtained the Bayes factors using bridge sampling, with a function built in the brms package. For example, to get the evidence in favor of the effect of Viewing time on item placement, we compare the full model (which includes Viewing time as a predictor) against a reduced model, which does not include Viewing time as predictor.

Specifically, we conducted a logistic regression using the Bernoulli likelihood function (with the default logit link function) to fit the models, since the outcome is a binary value (i.e., an item is placed or not). We specified the priors for the fixed effects’ as standard normal distribution (i.e., a normal distribution with a mean of 0 and a standard deviation of 1) (Gelman et al., 2017). If not explicitly mentioned we used the default settings of brms.

**Table 1:** Table of raw data points. This is an illustration of the structure of the data from a trial in a copying task. Each row represents the information (viewing time and delay) and fate (whether it is placed or not) of an item that is inspected during a viewing session. Note that these values match with the schematic overview of the measures shown in Fig. 3

Item	Viewing time (s)	Delay (s)	Placed
A	0.3	8.5	No
B	0.5	4.5	Yes
C	1.2	5.3	Yes
D	0.6	6.5	No

Unfortunately, the brms package does not provide a way to set a seed such that the exact output can be reproduced. Finally, note that we conform to the labels suggested by Kass and Raftery (1995) for the interpretation of Bayes factors (which were adapted from (Jeffreys, 1998)).

### Results

Our primary interest was how viewing time and delay affect VWM performance. But to get a general sense of how participants approached the task, we first report the summary statistics of participants’ behavior in the task. Participants took on average 44 (SD = 7.6) s to complete a single trial (i.e., copying one arrangement of eight items). On average, participants needed 6.3 (SD = 1.4) viewing sessions per trial, and took 12 (SD = 3.9) s per trial to view items. Finally, participants made on average 0.85 (SD = 0.57) errors per trial. These basic behavioral measures were comparable to previous (online) studies employing a similar task from our lab (Sahakian et al., 2023, 2024).

**Fig. 4:**
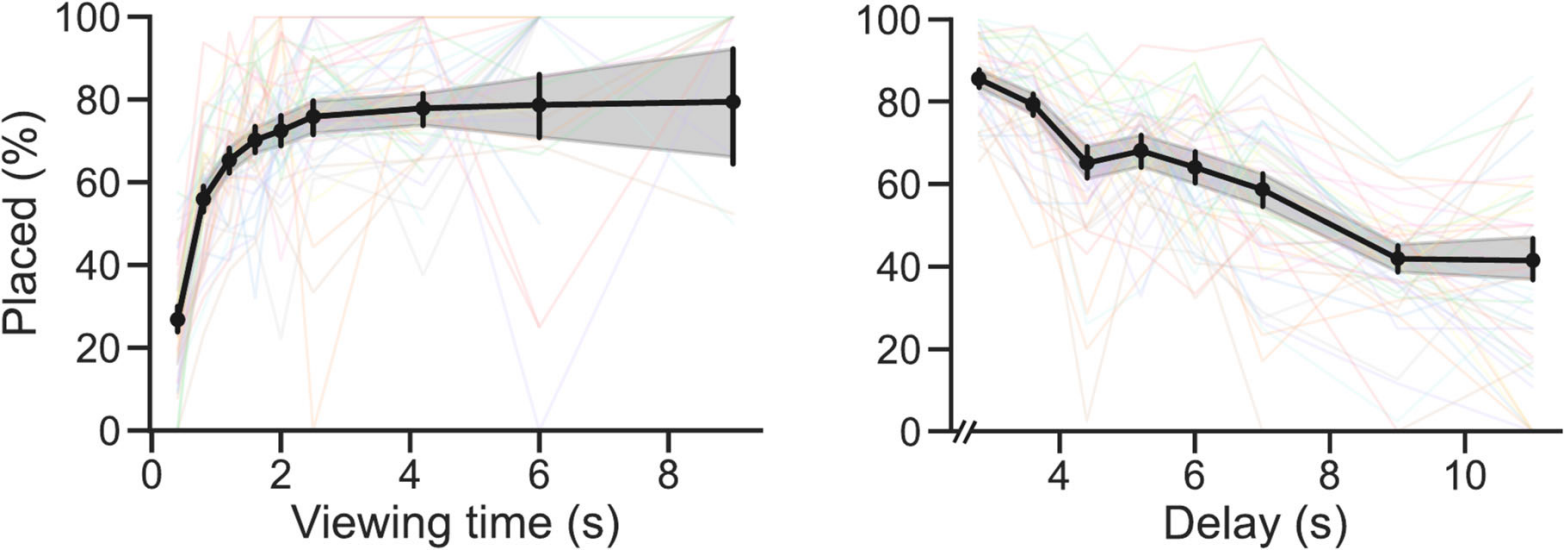
VWM performance in Experiment 1. The *y*-axes represent the memory performance in the copying task. More specifically, it shows the percentage of cases in which an item that was viewed was subsequently placed in the correct grid cell. On the left, the performance is shown over increasing viewing times. Each *point* represents a bin, as viewing times had a continuous spread. On the right, the performance is shown over increasing delays. A delay is the time an item is no longer viewed until the item is placed, or until a new viewing session begins. Again, each point represents a bin, as the delays also had a continuous spread. Each *colored line* in the background represents a participant’s mean values. The *error bars* and *shaded areas* represent the 95% confidence intervals of the participant means

**Table 2:** Logistic regression coefficients of the full model in Experiment 1. l-95% CI and u-95% CI represent lower and upper 95% credible intervals, respectively

	Estimate	Est. Error	l-95% CI	u-95% CI
Intercept	0.57	0.14	0.31	0.85
Viewing time	0.60	0.13	0.37	0.86
Delay	-0.34	0.03	-0.40	-0.29
nth Trial	0.04	0.00	0.03	0.04
nth Viewing session	0.07	0.01	0.05	0.09
n Item views	0.05	0.02	0.01	0.10
Viewing time X Delay	0.07	0.02	0.04	0.11

Regarding the temporal dynamics, we find decisive evidence in favor of including the effect of Viewing time in modeling item placement ($BF_{incl}\,=\,2.08\times 10^{115}$; see Fig. 4 and Table 2). The data showed that when an item was viewed longer, it was more likely to be placed correctly: the log-odds of placing an item increased by 0.60 (95% credible interval (CI) = [0.37, 0.86]) per additional second of (cumulative) viewing time.

To facilitate interpretation, we provide an example. First, we can raise *e* to the power of the log-odds to get an odds ($e^{\,log{\text {-}}odds}\,=\,odds$). As stated, the results show that for every additional second, an item is viewed the log-odds of placing an item increase by 0.60. Equivalently, we can state that every additional second an item is viewed, the odds of placing an item are increased by a *factor* of $e^{0.60}\,\approx \,1.82$. So if at some point, one had the odds of 0.25 (i.e., a probability of $\frac{0.25}{1 + 0.25} = 0.2$) to place an item correctly, looking for an additional second would increase the odds by a factor of 1.82, making the new odds equal $0.25 * 1.82\,\approx \,0.46$ (i.e., a probability of $\frac{0.48}{1 + 0.48}\,\approx \,0.31$)

Furthermore, we find decisive evidence in favor of including the effect of Delay in modeling item placement ($BF_{incl}\,=\,\infty$). The data showed that when the delay was longer, an item was less likely to be placed correctly ($\beta \,=\,-0.34$; 95% CI = [-0.40, -0.29]). To briefly illustrate using the previous example, if at some point one had the odds of 0.25 (a probability of 0.2) to place an item correctly, an additional second of delay means multiplying the odds by $e^{-0.34}\,\approx \,0.71$, making the new odds equal $0.25 * 0.71\,\approx \,0.18$ (a probability of $\frac{0.18}{1 + 0.18}\,\approx \,0.15$).

Finally, we find decisive evidence in favor of including the interaction effect of Viewing time and Delay in modeling item placement ($BF_{incl}\,=\,6.23\times 10^{8}$; $\beta \,=\,0.07$; 95% CI = [0.04, 0.11]). The data showed that for every additional second of Viewing time, the $\beta$ of Delay increases by 0.07. In other words, with increasing Viewing times, the effect of Delay became less detrimental.

**Fig. 5:**
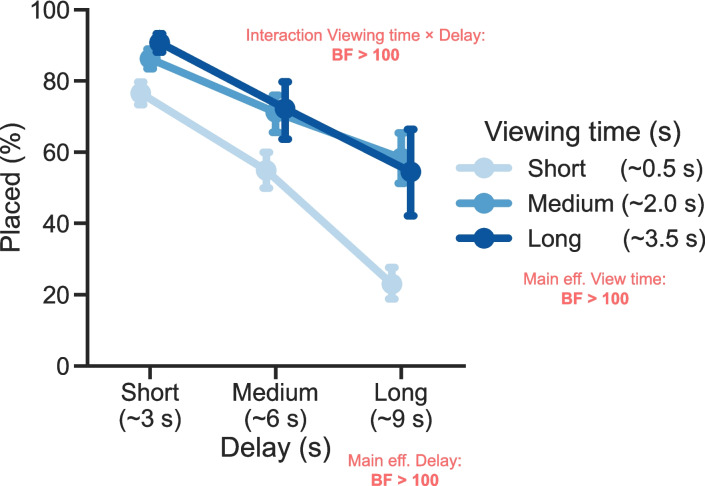
VWM performance in Experiment 1 across both viewing times and delay. The *y*-axes represent the memory performance in the copying task. More specifically, it shows the percentage of cases in which an item was viewed, and a successful action followed (i.e., placing the item in the correct grid cell). Each *point* represents all values within a range of viewing times and a range of delays. Viewing times were duration an individual items was viewed. The delay was the time from the point an item was no longer viewed until the item was placed, or until a new viewing session began. The *error bars* represent the 95% confidence intervals of the participant means

Although binning data depends on arbitrary choices, it does make it easier to visualize general patterns (see Figs. 5 and 7). So, to visualize the VWM representation strength across viewing time and delay, we binned all listed items (across all participants and all trials) into nine bins based on the items’ viewing times (short, medium, and long) and their delays (short, medium, and long).

In each of the nine bins, we computed the proportion of correctly placed items, by dividing the number of correctly placed items in the bin by the total number of items in the bin (i.e., the sum of correctly placed items and the not placed items).

## Interim discussion

In Experiment 1, we investigated how the time spent viewing visual information, and the time this information was retained before being used, jointly impacted memory performance. Importantly, we did so in a task that naturally engages VWM, and does not enforce any specific viewing or memorization strategies. Relating the independent measures of viewing time and delay to the memory performance showcases three distinct findings. First, in line with studies manipulating presentation times of memory arrays, the memory performance initially increases with longer viewing times but performance plateaus quickly, which means that from a certain encoding duration onward, there is no direct benefit of viewing any longer. Second, in line with studies manipulating delay periods, memory performance decays quickly after presentation ends, which means that it is advantageous to utilize memorized information as quickly as possible. Third, and arguably most interesting, we show there is an interaction between viewing time and delay on memory performance: after short viewing times, performance decays faster than after longer viewing times, which means that if you need to retain information for a long time it pays to look for longer.

Intrigued by this finding, we sought to further investigate the interaction between viewing time and delay. Before trying to pick apart which aspect of the naturalistic task caused the interaction to emerge, we aim to answer a more fundamental question first: "Is the observed interaction between viewing time and delay inherent to VWM?". If so, the interaction should be present in all paradigms, including the very basic paradigms, employed in VWM research. To the best of our knowledge, however, this interaction has not been reported in the VWM literature before.

**Fig. 6:**
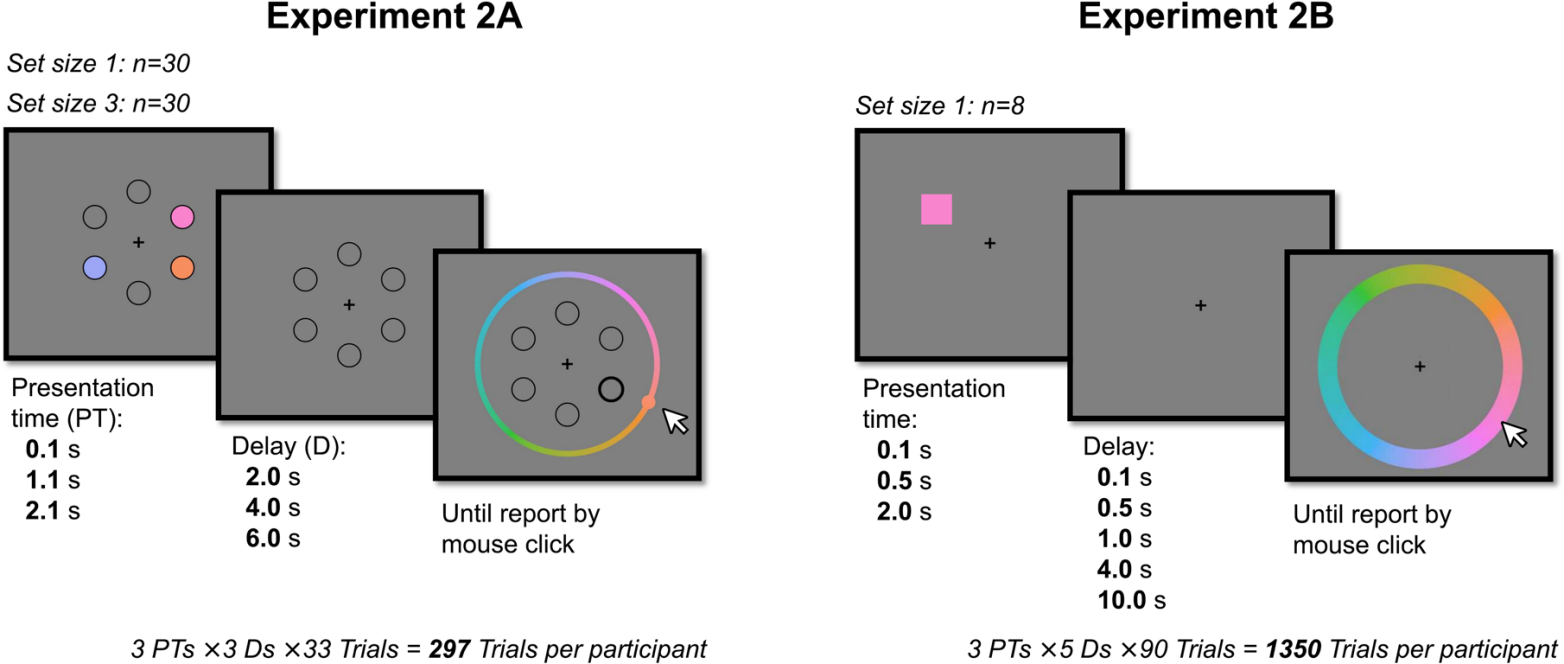
Trial overviews of Experiment 2A and Experiment 2B. In Experiment 2A (*left*) participants had to memorize either three (as displayed here) or one colored circles distributed over six possible locations. The colored circles were presented for 0.1, 1.1, or 2.1 s. After a delay of either 2, 4, or 6 s during which the colors were not visible, a color wheel appeared and the probed location was made bold. Participants had to report the color of the probed item by clicking on a specific hue on the color wheel. In Experiment 2B (*right*) participants had to memorize the color of a single square. The square could appear on the perimeter of a virtual circle around the fixation cross. The colored square was presented for 0.1, 0.5, or 2 s. After a delay of either 0.1, 0.5, 1, 4, or 10 s during which the square was not visible, a color wheel appeared. Participants had to report the color of the square by clicking on a hue on the color wheel. Please note that the specific stimulus features in this figure panel (sizes, colors, locations, etc.) do not exactly match those of the actual experiment. For the precise methods, we refer to the methods section of Donkin et al. (2015)

So, to address the fundamental question we employed a typical (i.e., temporally rigid) VWM task. We chose to base our paradigm on a continuous color report task used in Schurgin et al. (2020), as they demonstrated a presentation time and delay effect on memory strength using this paradigm. Moreover, a *continuous*, compared to a discrete trial paradigm is preferable, as each report provides more information about the memory strength (i.e., a continuous distance value to the true color, rather than a binary correct/incorrect). Crucially, though, Schurgin et al. (2020) manipulated presentation times *separately* from delay periods, making it impossible to investigate the aforementioned interaction. We therefore implement this paradigm, but manipulated both presentation times *and* delay periods simultaneously, in a within-participants design.

## Experiment 2A: A typical rigid VWM task

### Methods

#### Participants

We recruited participants via Prolific (www.prolific.co), a platform for recruiting participants for online studies. Using Prolific’s screening tool, we made our study available to participants who (1) reported to have no issue seeing colors, (2) were fluent in English, (3) were aged 18–35, (4) were located in the UK, and (5) had an approval rate higher of at least 95%. Upon successful completion of the experiment, participants received 5 GBP.

The experiment was designed and conducted in compliance with the Declaration of Helsinki, and it was approved by the Ethics Committee of the Faculty of Social and Behavioural Sciences of Utrecht University. The approval is filed under number 21-0297. All participants gave informed consent to use their data for scientific purposes.

We incrementally recruited participants for the online study until reaching the predetermined target of 60 participants. In total, data from 66 participants were collected, with six being excluded from the formal analysis due to incomplete data, either from early withdrawal or technical failures in saving the online data correctly.

#### Apparatus and stimuli

The task was generally based on the "Continuous colour report" task described in Schurgin et al. (2020). We coded the task and hosted it in Gorilla (Anwyl-Irvine et al., 2020), using the jsPsych (version 7.3) (de Leeuw, 2015) and the custom plugin "jspsych-psychophysics" (Kuroki, 2021).

We selected 360 colors from the HSLuv color space (www.hsluv.org). The hue values ranged from 0 up to 359 in steps of 1, the saturation value was fixed at 90, and the luminance value was fixed at 70. We ensured that any two colors presented in the same trial were more than 15 hue values separated from each other.

The memory items were colored circles with a radius of 32 pixels Fig. 6. They could appear at one of six fixed placeholder locations at an eccentricity of 140 pixels from a central fixation cross.

#### Procedure and design

After being presented the instructions and completing four practice trials, participants completed the experimental trials. In each trial, a participant was presented with either 1 or 3 items, then after a delay, one of the items’ locations was emboldened, prompting the participant to report the color of the indicated item (see Fig. 6). Participants could report the color on a continuous color wheel (specifically, 360 options), using their mouse. A ball moved around the color wheel as the cursor moved around the central fixation cross. The ball had the color of that part of the color wheel it was on, indicating the current selected color. Participants moved their mouse to select a color and confirmed their report by clicking.

We manipulated our two factors of primary interest, presentation time and delay, within participants, but we manipulated set size between participants. The memory items were presented for either 0.1, 1.1, or 2.1 s, and were then removed for a delay period of 2, 4, or 6 s. Half of the participants were presented with one item in each trial, and the other half with three items. For each of the nine combinations of a presentation time and delay, there were 33 trials, resulting in 297 trials per participant. The order of the trials was randomized.

Due to a randomization error, for each participant only a set of nine unique trials (comprising all combinations of the three presentation times and three delay conditions) was generated and presented. This entailed that in all cases where the presentation time was 1.1 s and the delay 6 s, the same set of colors in the same locations was presented and the same items was probed. This held for each of the nnine unique combinations of presentation times and delays. All participants, therefore, repeated each of their nine trials 33 times in an intermixed fashion.^2^

#### Analysis

We performed a Bayesian linear mixed models analysis using the BayesFactor package in R (Morey & Rouder, 2024; R Core Team, 2024). The dependent variable was the precision of the reports on the color wheel. Precision was computed as the reciprocal of the circular standard deviation, minus the expected value if all responses would be purely random (Bays et al., 2011). As such, a precision of 0 corresponds to random guesses, and higher values correspond to qualitatively better reports. The three independent variables were presentation time, delay (manipulated within participants) and set size (manipulated between participants). Specifying participant as a random effect, we tested all possible models against the intercept-only model. To quantify the evidence of a (main or interaction) effect, we computed the inclusion Bayes factor across matched models only, using the R package bayestestR (Hinne et al., 2020; Makowski et al., 2019; Mathôt, 2017). We used the default settings for the analysis both R packages (notably the default priors are specified as a Cauchy with scale 0.5 for fixed effects, and a Cauchy with scale 1 for random effects), and we set the seed value to 1 in R for reproducibility.

### Results

**Fig. 7:**
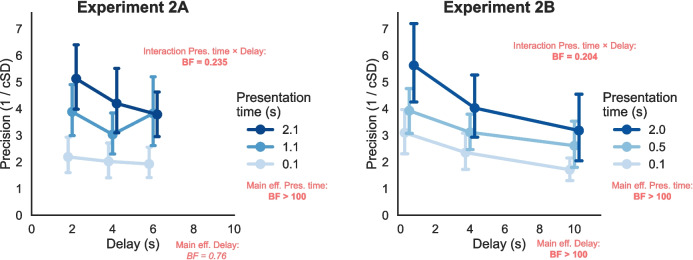
Precision of responses in Experiment 2A and Experiment 2B. Note that the data of Experiment 2A (*left*) is the pooled data of the set size one and set size three conditions (see Supplementary material C for results split by set size). Also note that in Experiment 2B (*right*) there were five delays, but for the sake of clarity of the visualization, we pooled the data of the 0.1-, 0.5-, and 1-s delay conditions (which had very similar precision). The precision of the responses was computed as the reciprocal of the circular standard deviation (1/cSD) of the errors, minus the expected value if all reports would be random. The *error bars* represent the 95% confidence intervals of the participant means

We found decisive evidence for a main effect of presentation time ($\beta$ = 1.12; 95% CI = [0.83, 1.39]; $BF_{incl}\,=\,1.48\times 10^{12}$. This means that for every additional second an item was presented, the precision increased by 1.29 (see Fig. 7). We found no conclusive evidence for a main effect of delay ($\beta$ = -0.13; 95% CI = [-0.271, 0.003]; $BF_{incl}\,=\,0.76$). This entails that we cannot draw conclusions about the effect of delay on the precision of the responses. We found strong evidence for a main effect of set size ($\beta$ = -0.79; 95% CI = [-1.38 -0.208]; $BF_{incl}\,=\,24.18$). This means that for every additional item that was presented the precision per item decreased by 0.79. Furthermore, we found substantial evidence against an interaction effect of presentation time and delay ($\beta$ = -0.13; 95% CI = [-0.30, 0.04]; $BF_{incl}\,=\,0.235$). This entails that the presentation time does not change the effect of delay on the precision of the responses. Similarly, we found evidence against the other two-way and the three-way interactions. Set size $\times$ Presentation time: $BF_{incl}\,=\,0.079$, Set size $\times$ Delay: $BF_{incl}\,=\,0.197$, Set size $\times$ Presentation time $\times$ Delay: $BF_{incl}\,=\,0.215$ (see Supplementary material C for results split by set size).

## Interim discussion

In a typical, temporally rigid VWM task, we parametrically manipulated the presentation times of memory items and of the delays until a forced memory report. Under these task circumstances, we found evidence against an interaction between presentation time and delay on VWM performance. Even though the main effect of presentation time was evidently present, the main effect for delay was much less so. The lack of an effect for delay might therefore hide the potentially present interaction effect between presentation time and delay. Possibly, our experiment contained too few trials per delay condition (99 versus 300 in the experiment by Schurgin et al., 2020) to produce a reliable effect of delay. Our choice of the number of trials per condition was limited by the maximum duration of the online experiment. Fortunately, a published study by Donkin et al. (2015), employed a paradigm quite similar to Experiment 2A of this study. Although their experiment aimed to answer very different question, it was also suited to answer our question: whether, in a temporally rigid VWM task, presentation times determined the rate of decay of VWM representations.

## Experiment 2B: Reanalysis of Donkin et al. (2015)

### Methods

For the full methods, we refer to the original article (Donkin et al., 2015). For our current purposes, we describe only the relevant sections. Generally, this paradigm is very similar to the set size 1 condition of Experiment 2A. On standard trials^3^ participants were presented with one colored square for a variable presentation time, and after a variable delay period, reported the specific hue of the square on a continuous color wheel.

There were three presentation times: 0.1, 0.5, and 2 s, and there were five delays: 0.1, 0.5, 1, 4, 10 s. Each combination of presentation time and delay occurred 90 times for each participant in a randomly intermixed order. Eight participants completed this study in a lab.

#### Analysis

The analysis was almost identical to that of Experiment 2A. The only difference was that we could not consider a set size effect, as the set size was always one in this study.

### Results

We found decisive evidence for a main effect of presentation time ($\beta$ = 1.03; 95% CI = [0.79, 1.26]; $BF_{incl}\,=\,1.48\times 10^{11}$). This means that for every additional second an item was presented, the precision increased by 1.03 (see Fig. 7). We found decisive evidence for a main effect of delay ($\beta$ = -0.20; 95% CI = [-0.26, -0.15]; $BF_{incl}\,=\,2.09\times 10^{9}$). This entails that for every additional second of delay, the precision of the responses decreases by 0.20. Finally, we found substantial evidence against an interaction effect of presentation time and delay ($\beta$ = -0.06; 95% CI = [-0.13, 0.001]; $BF_{incl}\,=\,0.204$). This entails that the presentation time does not change the effect of delay on the precision of the responses.

## Interim discussion

Using a previously published dataset, we aimed to find whether longer presentation times for visual information result in a slower decay of VWM representations, akin to the findings of Experiment 1. Importantly, we were now specifically interested in whether this was the case in rigid VWM tasks. In the reanalysis of Donkin et al. (2015) we found very strong evidence for both an effect of presentation time, and of delay. Yet, we still found evidence against an interaction between presentation time and delay. Finding evidence against an interaction in two separate (tightly controlled) VWM experiments further corroborates the idea that the interaction we found in Experiment 1 of the current study is not an inherent aspect of VWM.

## General discussion

In this study, we investigated how self-paced viewing times and delays jointly determine how memories are formed and how they fade. Although the time courses of memory buildup and memory decay have been studied before, they have mainly been studied separately. What is more, they have exclusively been studied in temporally rigid experimental designs. These rigid paradigms have been unable to capture an essential aspect of naturalistic working memory use: in almost all our daily memory-guided behavior, our interactions are self-paced, rather than enforced on us. This self-paced nature of our interactions might elicit a very different behavior than researchers have measured thus far in the lab. To fill this gap, we employed a copying task, in which participants recreated an (always accessible) example arrangement of items in an empty space. In this task, participants were not restricted in the choice or number of items to memorize or report, nor in the amount of time spent encoding or retaining these items in memory before placing them on the workspace grid. By using such an unrestricted visual working memory (VWM) task, we evoked a wide range of self-paced viewing times and delays, much like one would encounter in everyday human behavior. With the current paradigm, we replicated two main findings from previous literature (Bays et al., 2011; Brady et al., 2016; de Jong et al., 2023; Donkin et al., 2015; Oberauer & Lewandowsky, 2008; Souza & Oberauer, 2015; Souza et al., 2016; Zhang & Luck, 2009): (1) an initial increase in memory performance, which eventually plateaued as viewing times increased beyond a second, and (2) a monotonic decrease in memory performance as delays increased.

Importantly, we found an interaction between viewing time and delay on memory performance that has not been reported before in the short-term memory literature: as viewing times increased, the rate of decay became slower. This interaction is reminiscent of the age-old adage of teachers: "If you shortly study for an exam the night before, you might pass the next day but you will have forgotten everything the next week. But when you study for longer, you will pass the exam and you will likely remember what you learned for the years to come." We therefore subsequently hypothesized that the interaction might be a fundamental attribute of working memory representations – this claim has been made after all for long-term memory representations (Yang et al., 2016). Note that before teasing apart which of the many naturalistic aspects of the task contributed to the interaction, we aimed to verify the more fundamental hypothesis first. To our surprise, however, one experiment (Experiment 2A) and one reanalysis of a previously published study (Experiment 2B) – both using typically rigid paradigms – showed converging evidence that the precision of visual working memory representations do *not* follow the same pattern: The precision of representations degraded at the same rate regardless of the time visual information was presented. This leads us to conclude that the interaction we find using a naturalistic paradigm does *not* reflect a fundamental attribute of VWM representations. In the light of our previously proposed model describing naturalistic VWM use (Sahakian et al., 2023), we discuss four distinct mechanisms that could potentially reconcile the emergence of an interaction in the naturalistic VWM task and an absence hereof in rigid force-choice experiments. We first briefly explain our model, and then discuss the four explanatory mechanisms.

**Fig. 8:**
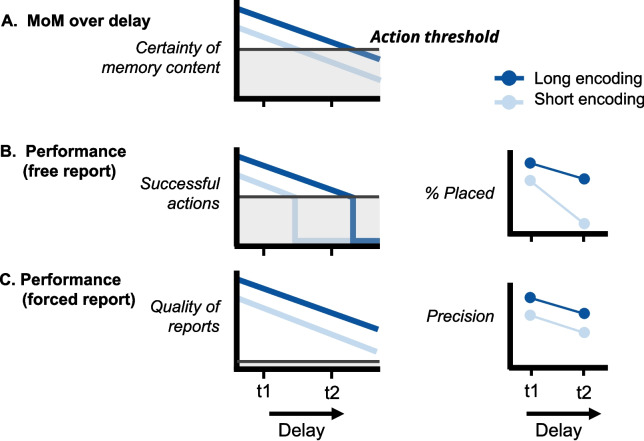
The emergence of an interaction due to the nature of a task. This schematic shows how an interaction can emerge between the effect of viewing times and the effect of delays on performance in free report paradigms (like the copying task in Experiment 1), while there is no interaction in equivalent forced report paradigms (like the typical VWM tasks in Experiment 2A and 2B). **A** Illustration of how two traces of certainty decline gradually over a delay period. The *dark blue line* shows the certainty of an item that was viewed for longer and thus had a higher certainty to begin with. The *light blue line* shows the certainty of an item that was viewed for shorter. The *marked* and *shaded area* represent the "Action threshold": when the certainty of a memory drops below the line, it will not be acted upon. **B** The decline of performance in a free report paradigm. Performance in such tasks is generally measured by whether a successful action took place. At the two time points where the respective certainties drop below the action threshold, no actions take place, and as such, the performance measure for "successful actions" drops to zero. **C** The decline of performance in a forced report paradigm. Performance in such tasks is generally measured by the quality of the reports (e.g., precision, accuracy, etc.). As the responses are forced, the Action threshold becomes irrelevant (or more accurately, in forced-choice tasks the Action threshold is lowered to zero), and performance tracks certainty of the memories. Note that the *light gray shaded areas* in the bottom two plots are only there to help marking the moments at which the certainties would drop below the action threshold, and should not be interpreted as the action threshold (as the action threshold is measured in certainty, not in performance). The two smaller plots on the right show the performance of the two types of tasks (free vs. forced report) at two delays (t1 and t2). The *y*-axis labels mimic the performance measures of Experiment 1, and Experiment 2 (A & B), respectively. In the upper right plot (representing a copying task), there is an interaction between viewing time and delay, while in the lower right plot (representing a typical VWM task) there is no interaction

In previous studies, we introduced and expanded the Mountains of Memory (MoM) model of VWM use in day-to-day behavior (Sahakian et al., 2023, 2024). In short, this model describes whether an observer will choose to utilize memory content or re-examine the external world instead. This decision is determined by the certainty (or knowledge about the quality) of the VWM content and a context-dependent action threshold. The certainty of the memory content can be thought of as a mountainous landscape, where different stimulus features (i.e., color, shape, etc.) exist in different locations of the landscape, and the height of the peaks at each location describes the amount of certainty for the memory representation of that feature; the higher the peak, the more likely an observer is to (correctly) report the corresponding feature. In this analogy, the action threshold, thought of as the height of a sea inundating the landscape, determines what memory content is deemed good enough to act on in a given situation (i.e., parts of the landscape above sea level). In our work so far, we have used this model to describe differences in VWM use between conditions (e.g., a condition in which actions are penalized more severely than another, thus increasing the threshold for action). Importantly, in this earlier work, we considered the certainty of the representations only at a single point in time: the (virtual) moment at which a decision is made. In the current study, which considers the whole time range, the findings highlight the sometimes counter-intuitive influence of an action threshold on VWM use, when memory representations decay over time. Specifically, we postulate that the simple concept of an action threshold (which determines whether or not observers will act upon VWM content) can explain the observed interaction of memory performance between viewing time and delay in VWM use, even if VWM representations themselves would build up and decay in a non-interactive manner.

The first mechanism that might explain the emergence of an interaction in naturalistic VWM task, is that actions are never forced in the naturalistic VWM task, while in the rigid task actions are always forced (we provide a visual explanation in Fig. 8). In the copying task, participants repeatedly choose whether to act on a memory (i.e., pick up an item and place it somewhere). In some cases (when confident of their memory) they might try to place an item, in other cases (when unsure of their memory) they might choose not to place an item but have a look at the example again. In our MoM model, the *Action threshold* indicates the minimal required certainty to act on memory content. Indeed, previous work has shown that when participants look at the example again, memory is not fully depleted, as participants have some residual (albeit unsure) memories of items (Sahakian et al., 2023). In the copying task, not acting on a memory that is there (because the certainty does not exceed the Action threshold) leads to a worse performance measure: we register that no successful action followed an item view. Compare this to not filling in an answer in a multiple-choice test on questions you are unsure of your answer: Not filling in an answer, likewise, leads to a worse performance measure on the test (compared to always filling in your best guess). In contrast, in typical VWM tasks, actions (or responses) are forced (akin to an action threshold that is infinitely low): Participants provide their best guess every time the memory is probed.

For forced report designs, this means that with decreasing representation strength (e.g., due to increasing delays), the outcome measure gradually decreases. In free report designs, however, with decreasing representation strength (or certainty), the outcome measure also gradually decreases until the certainty falls below the Action threshold. From that point onward, the outcome measure drops sharply to zero, as no actions are undertaken. This non-linear relation between memory decay and the outcome measure in free report paradigms (such as the copying task) give rise to an interaction because the action threshold is reached later when the initial memory certainty was high and is reached earlier when the initial memory certainty was low (see Fig. 8).

The second possible reason is the amount of interference during the delay period (Oberauer & Lin, 2023). While in the continuous recall tasks there is only a blank screen to look at, in the copying task, participants see and do much more in the delay period (i.e., memorize other items, search for and place items). Perhaps, knowing that there will be visual interference and other mentally demanding actions, prompts participants to encode the task-relevant information much better, in anticipation of various distractions. Therefore, specifically in the naturalistic task, participants might use longer viewing times to generate more robust representations which remain strong enough to use after prolonged periods. In typical paradigms, there is less interference and the interference is predictable and uniform across conditions (Bays et al., 2011; Schurgin et al., 2020). A predictable and uniform interference in all conditions (common in typical VWM tasks) likely encourages a uniform encoding strategy in all conditions.

The third possible reason is that longer viewing times perhaps protect representations from output interference (Cowan et al., 2002; Liu & Caplan, 2020). Output interference refers to the degradation of memory contents due to retrieving and acting on (part of) the contents. It is safe to assume that items that are placed later in a sequence of placements, have longer delay times. As such, these later placed items are more susceptible to interference caused by actions preceding their placement. Output interference is a striking example of the differences between everyday VWM use and the typical paradigms employed in VWM research: while making multiple actions after a single look is common in daily behavior, most VWM tasks require only a single report for each memorized display.

Finally, the fourth possible reason is the number of rehearsals. In Experiment 1, this translates to the number of times an item was viewed. Although the crucial interaction was present despite controlling for the number of views, we did not rule out that the number of views might contribute to the interaction. Besides, the possibility of rehearsals is a pronounced difference between typical and self-paced VWM tasks (and indeed between everyday VWM use).

Indeed, there are many other differences between Experiment 1 and Experiments 2A & 2B, which we do not address to keep focus. In any case though, we think the findings of Experiments 2A and 2B allows us to conclude that this key interaction (i.e., between viewing time and delay on memory performance) is *not* a fundamental principle of visual working memory.

Regarding the paradigm of the main task (i.e., the copying task in Experiment 1), it is worth noting why the self-paced behavior is so relevant in making this task more naturalistic. Almost all prior studies devoted to temporal dynamics of VWM enforce a preset temporal structure (e.g., several presentations times, or a few forced delay durations) (Bays et al., 2011; Donkin et al., 2015; Oberauer & Lewandowsky, 2008; Rademaker et al., 2018; Schurgin et al., 2020). Although this approach might give good estimates of the VWM performance at the time points being probed, there is a drawback as well: Especially for longer presentation times, it might be unclear whether participants are attentively encoding the visual stimulus for the entire period it is presented. Perhaps participants might feel after a short while that they encoded the stimulus well enough, and for the rest of the period they stop encoding. After all, in natural viewing conditions humans make about three eye movements per second, meaning they keep their gaze at one point generally for only a few hundred milliseconds (Henderson, 2003). VWM is therefore not attuned to work in the context of being forced to look at objects for a predetermined period of time. In the current copying task, participants determine themselves how long they fixate a stimulus, which is far more in line with the everyday use of VWM. Moreover, the viewing time probably reflects the true encoding duration better, under the assumption that participants stop looking when they are done encoding.

Despite the obvious advantages of the self-paced nature of our main task, this design choice also obscured some interesting aspects of the buildup and decay of VWM content. In our analyses, we did not consider the events that happened during the delay period. A few examples of what could happen during the delay are: (1) other items might be viewed, thus loading up VWM even more, (2) other items might be placed, thus activating and acting on other VWM content, or (3) time might be spent on deciding what to do, thus increasing the delay duration. Of course, all these processes are not mutually exclusive. As such, the contribution of these individual processes to the decay of VWM representations is hard to disentangle. Future research into the role of viewing time and delay on VWM-based actions will shed more light on the temporal aspects of the buildup and decay of memories.

From a broader perspective, the time course of the buildup and decay of visual memories is likely related to the way information is gathered: how long objects are inspected, and if (and when) are they *re*inspected? It has been previously argued that the amount of information humans internalize (i.e., encode into working memory) versus the amount they leave in the external world for later internalization depends on a trade-off between the costs associated with internalizing and the costs associated with leaving information in the external world (O’regan, 1992; Somai et al., 2020; Van der Stigchel, 2020). In this framework, how long information is looked at can be regarded as a cost associated with internalizing information. In the context of such a trade-off, the time invested in viewing information is an important factor: looking for longer presumably strengthens the memory, but depending on the context the amount of time available might be limited. Furthermore, as viewing time increases, the added benefit of viewing for longer diminishes.

Having an understanding of how the viewing duration determines how well information is stored can reveal what the optimal course of action is in the trade-off between internalizing information or leaving it externally. In the same vein, how quickly information fades away from VWM might affect this trade-off as well: if one needs to retain information for a prolonged time, it is worth to invest more time in looking. Studying temporal dynamics in naturalistic contexts can thus open new avenues for instructing people with memory deficits to better deal with their impairment. Beyond clinical relevance, these findings can also be relevant for professionals doing VWM-demanding work under time pressure (e.g., air traffic controllers, radiologists). An important step we advocate for future research is to make explicit which aspects of naturalistic VWM use (e.g., self-pacedness, rehearsals, output interference), are incorporated in one’s experimental design and analysis, and which aspects are not.

In sum, we investigated how the time spent looking at visual information and the time that this information was kept in VWM jointly determine how memories are put to use. By employing a self-paced, naturalistic VWM task, we replicated two key findings of typical VWM studies, and revealed a third and novel finding: (1) with increasing viewing time, performance gets better until it plateaus; (2): performance deteriorates with increasing delays, and (3) we uniquely show that in a naturalistic task, performance deteriorates slower following longer viewing times. We also showed that a slower deterioration of performance after longer viewing times is *not* a fundamental property of VWM. Rather, we argue, it is the naturalistic context of the task, which gives rise to this pattern of more robust memories with longer viewing times. We encourage future research to further elucidate which factors contribute to this interesting pattern of results in naturalistic VWM use. With this research, we bridge the gap between the way memories themselves develop over time and how they relate to memory-based actions.

## Supplementary Information

Below is the link to the electronic supplementary material.

Supplementary file 1 (pdf 232 KB)

Supplementary file 2 (pdf 211 KB)

Supplementary file 3 (pdf 46 KB)

## Data Availability

All data (both raw and processed) are uploaded on the OSF platform, and are accessible via the link: https://osf.io/dg73u/.

## Footnotes

1 By this procedure instances where the aperture moved across an item’s cell were also labeled as (very short) views. To remove such cases, we implemented an additional rule that only views which were longer than 120 ms were labeled as views. The threshold of 120 ms was chosen as the most reasonable value based on the distribution of all viewing times. We realize that this is an arbitrary choice, and therefore we performed our main analyses with other thresholds that were within a reasonable range (specifically: 60, 80, 100, 140, 160, 180 ms). We found that all options produced equivalent results. We report these results in Supplementary material A.

2 In principle, participants could learn which item in a memory array would be probed, but an additional analysis (see Supplementary material B) showed that performance did not substantially increase over the 33 repetitions of a trial. This indicates that participants likely did not notice nor benefit from this unintended randomization procedure.

3 There were also so-called "labeling" trials interspersed with the standards trials, but these are irrelevant for our purposes, so we ignore them in our discussion of the study.

## Code availability

All data processing scripts and statistical analyses are uploaded on the OSF platform, and are accessible via the link: https://osf.io/dg73u/.

## Declarations

### Open Practices Statement

The data and materials for all experiments are available at: https://osf.io/dg73u/. None of the experiments was preregistered.
