# Supplementary material for: The rise and fall of memories: Temporal dynamics of visual working memory
Source: Mem Cognit. 2025 May 6;53(8):2406–23. doi: 10.3758/s13421-025-01718-9 (PMC12695987; doi:10.3758/s13421-025-01718-9)
Supplement: Supplementary file 1 — (pdf 232 KB) [file 13421_2025_1718_MOESM1_ESM.pdf]

Supplementary material A

Below we report the model summaries for the GLMM fits for a range of reasonable cutoff values for including item views. Specifically we report the summaries for cutoff values: 60, 80, 100, 140, 160, 180 ms.

Cutoff = 60 ms

```
Generalized linear mixed model fit by maximum likelihood (Laplace Approxima
tion) ['glmerMod']
 Family: binomial  ( logit )
Formula: placed ~ cumul_view_time * delay + trial_number + after_nth_view_s
es +      (1 + cumul_view_time * delay | id)
   Data: df

     AIC      BIC   logLik deviance df.resid 
 19373.7  19499.5  -9670.9  19341.7    19178 

Scaled residuals: 
     Min       1Q   Median       3Q      Max 
-157.545   -0.641    0.311    0.642   11.533 

Random effects:
 Groups Name                  Variance Std.Dev. Corr             
 id     (Intercept)           0.64757  0.80472                   
        cumul_view_time       0.81730  0.90404  -0.43            
        delay                 0.03311  0.18197  -0.29 -0.39      
        cumul_view_time:delay 0.00394  0.06277   0.18 -0.04 -0.75
Number of obs: 19194, groups:  id, 46

Fixed effects:
                       Estimate Std. Error z value Pr(>|z|)    
(Intercept)            0.384013   0.149887   2.562   0.0104 *  
cumul_view_time        0.884757   0.155173   5.702 1.19e-08 ***
delay                 -0.367893   0.030862 -11.921  < 2e-16 ***
trial_number           0.038887   0.002157  18.032  < 2e-16 ***
after_nth_view_ses     0.070831   0.009018   7.854 4.03e-15 ***
cumul_view_time:delay  0.076554   0.018477   4.143 3.42e-05 ***
---
Signif. codes:  0 ‘***’ 0.001 ‘**’ 0.01 ‘*’ 0.05 ‘.’ 0.1 ‘ ’ 1

Correlation of Fixed Effects:
            (Intr) cml_v_ delay  trl_nm aft___
cumul_vw_tm -0.513                            
delay       -0.420 -0.116                     
trial_numbr -0.241  0.028 -0.009              
aftr_nth_v_ -0.241 -0.005  0.019  0.154       
cml_vw_tm:d  0.379 -0.388 -0.664 -0.022 -0.014
optimizer (Nelder_Mead) convergence code: 0 (OK)
Model failed to converge with max|grad| = 0.0160274 (tol = 0.002, component
1)
```

Cutoff = 80 ms

```
Generalized linear mixed model fit by maximum likelihood (Laplace Approxima
tion) ['glmerMod']
 Family: binomial  ( logit )
Formula: placed ~ cumul_view_time * delay + trial_number + after_nth_view_s
es +      (1 + cumul_view_time * delay | id)
   Data: df

     AIC      BIC   logLik deviance df.resid
 19147.7  19273.2  -9557.9  19115.7    18782

Scaled residuals:
    Min      1Q  Median      3Q     Max
-90.524  -0.653   0.336   0.642  39.881

Random effects:
 Groups Name                  Variance Std.Dev. Corr
 id     (Intercept)           0.638683 0.79918
        cumul_view_time       0.654822 0.80921  -0.42
        delay                 0.031050 0.17621  -0.34 -0.35
        cumul_view_time:delay 0.004231 0.06505   0.23  0.01 -0.79
Number of obs: 18798, groups:  id, 46

Fixed effects:
                       Estimate Std. Error z value Pr(>|z|)
(Intercept)            0.467154   0.149753   3.120  0.00181 **
cumul_view_time        0.820546   0.143486   5.719 1.07e-08 ***
delay                 -0.358929   0.030070 -11.936  < 2e-16 ***
trial_number           0.037717   0.002166  17.409  < 2e-16 ***
after_nth_view_ses     0.071271   0.009083   7.846 4.28e-15 ***
cumul_view_time:delay  0.072939   0.018515   3.939 8.17e-05 ***
---
Signif. codes:  0 ‘***’ 0.001 ‘**’ 0.01 ‘*’ 0.05 ‘.’ 0.1 ‘ ’ 1

Correlation of Fixed Effects:
            (Intr) cml_v_ delay  trl_nm aft___
cumul_vw_tm -0.516
delay       -0.461 -0.056
trial_numbr -0.242  0.027 -0.011
aftr_nth_v_ -0.243 -0.005  0.019  0.154
cml_vw_tm:d  0.401 -0.394 -0.698 -0.015 -0.013
optimizer (Nelder_Mead) convergence code: 0 (OK)
Model failed to converge with max|grad| = 0.0198533 (tol = 0.002, component
1)
```

Cutoff = 100 ms

```
Generalized linear mixed model fit by maximum likelihood (Laplace Approxima
tion) ['glmerMod']
 Family: binomial  ( logit )
Formula: placed ~ cumul_view_time * delay + trial_number + after_nth_view_s
es +      (1 + cumul_view_time * delay | id)
   Data: df

     AIC      BIC   logLik deviance df.resid 
 18935.8  19060.9  -9451.9  18903.8    18383 

Scaled residuals: 
   Min     1Q Median     3Q    Max 
-32.57  -0.67   0.36   0.65 695.28 

Random effects:
 Groups Name                  Variance Std.Dev. Corr             
 id     (Intercept)           0.507921 0.7127                    
        cumul_view_time       0.525228 0.7247   -0.37            
        delay                 0.022771 0.1509   -0.25 -0.42      
        cumul_view_time:delay 0.002895 0.0538    0.03  0.10 -0.73
Number of obs: 18399, groups:  id, 46

Fixed effects:
                       Estimate Std. Error z value Pr(>|z|)    
(Intercept)            0.514354   0.139906   3.676 0.000237 ***
cumul_view_time        0.760876   0.132612   5.738 9.60e-09 ***
delay                 -0.343401   0.026650 -12.886  < 2e-16 ***
trial_number           0.036596   0.002173  16.843  < 2e-16 ***
after_nth_view_ses     0.071896   0.009134   7.872 3.50e-15 ***
cumul_view_time:delay  0.068108   0.017008   4.004 6.22e-05 ***
---
Signif. codes:  0 ‘***’ 0.001 ‘**’ 0.01 ‘*’ 0.05 ‘.’ 0.1 ‘ ’ 1

Correlation of Fixed Effects:
            (Intr) cml_v_ delay  trl_nm aft___
cumul_vw_tm -0.501                            
delay       -0.424 -0.060                     
trial_numbr -0.260  0.028 -0.012              
aftr_nth_v_ -0.261 -0.005  0.019  0.156       
cml_vw_tm:d  0.349 -0.398 -0.672 -0.014 -0.011
optimizer (Nelder_Mead) convergence code: 0 (OK)
Model failed to converge with max|grad| = 0.0329213 (tol = 0.002, component
1)
```

Cutoff = 140 ms

```
Generalized linear mixed model fit by maximum likelihood (Laplace Approxima
tion) ['glmerMod']
 Family: binomial  ( logit )
Formula: placed ~ cumul_view_time * delay + trial_number + after_nth_view_s
es +      (1 + cumul_view_time * delay | id)
   Data: df

     AIC      BIC   logLik deviance df.resid 
 18412.4  18536.9  -9190.2  18380.4    17651 

Scaled residuals: 
    Min      1Q  Median      3Q     Max 
-35.592  -0.693   0.390   0.639 287.363 

Random effects:
 Groups Name                  Variance Std.Dev. Corr             
 id     (Intercept)           0.405150 0.63651                   
        cumul_view_time       0.292333 0.54068  -0.20            
        delay                 0.027754 0.16659  -0.41 -0.36      
        cumul_view_time:delay 0.003643 0.06036   0.13  0.20 -0.85
Number of obs: 17667, groups:  id, 46

Fixed effects:
                       Estimate Std. Error z value Pr(>|z|)    
(Intercept)            0.671277   0.132122   5.081 3.76e-07 ***
cumul_view_time        0.590576   0.110645   5.338 9.42e-08 ***
delay                 -0.332910   0.028627 -11.629  < 2e-16 ***
trial_number           0.035204   0.002197  16.021  < 2e-16 ***
after_nth_view_ses     0.072299   0.009281   7.790 6.72e-15 ***
cumul_view_time:delay  0.071625   0.016897   4.239 2.25e-05 ***
---
Signif. codes:  0 ‘***’ 0.001 ‘**’ 0.01 ‘*’ 0.05 ‘.’ 0.1 ‘ ’ 1

Correlation of Fixed Effects:
            (Intr) cml_v_ delay  trl_nm aft___
cumul_vw_tm -0.450                            
delay       -0.510  0.020                     
trial_numbr -0.281  0.034 -0.009              
aftr_nth_v_ -0.280 -0.005  0.014  0.159       
cml_vw_tm:d  0.401 -0.413 -0.739 -0.015 -0.007
optimizer (Nelder_Mead) convergence code: 0 (OK)
Model failed to converge with max|grad| = 0.0462475 (tol = 0.002, component
1)
```

Cutoff = 160 ms

```
Generalized linear mixed model fit by maximum likelihood (Laplace Approxima
tion) ['glmerMod']
 Family: binomial  ( logit )
Formula: placed ~ cumul_view_time * delay + trial_number + after_nth_view_s
es +      (1 + cumul_view_time * delay | id)
   Data: df

     AIC      BIC   logLik deviance df.resid 
 18177.9  18302.0  -9072.9  18145.9    17282 

Scaled residuals: 
    Min      1Q  Median      3Q     Max 
-20.224  -0.710   0.408   0.639 267.329 

Random effects:
 Groups Name                  Variance Std.Dev. Corr             
 id     (Intercept)           0.402244 0.63423                   
        cumul_view_time       0.215314 0.46402  -0.20            
        delay                 0.020745 0.14403  -0.39 -0.38      
        cumul_view_time:delay 0.002668 0.05165   0.10  0.26 -0.85
Number of obs: 17298, groups:  id, 46

Fixed effects:
                       Estimate Std. Error z value Pr(>|z|)    
(Intercept)            0.704101   0.131986   5.335 9.57e-08 ***
cumul_view_time        0.529067   0.101602   5.207 1.92e-07 ***
delay                 -0.310155   0.025527 -12.150  < 2e-16 ***
trial_number           0.034771   0.002209  15.739  < 2e-16 ***
after_nth_view_ses     0.070623   0.009317   7.580 3.46e-14 ***
cumul_view_time:delay  0.064494   0.015527   4.154 3.27e-05 ***
---
Signif. codes:  0 ‘***’ 0.001 ‘**’ 0.01 ‘*’ 0.05 ‘.’ 0.1 ‘ ’ 1

Correlation of Fixed Effects:
            (Intr) cml_v_ delay  trl_nm aft___
cumul_vw_tm -0.469                            
delay       -0.515  0.072                     
trial_numbr -0.285  0.039 -0.007              
aftr_nth_v_ -0.282 -0.004  0.016  0.159       
cml_vw_tm:d  0.402 -0.446 -0.744 -0.021 -0.008
optimizer (Nelder_Mead) convergence code: 0 (OK)
Model failed to converge with max|grad| = 0.0505777 (tol = 0.002, component
1)
```

Cutoff = 180 ms

```
Generalized linear mixed model fit by maximum likelihood (Laplace Approxima
tion) ['glmerMod']
 Family: binomial  ( logit )
Formula: placed ~ cumul_view_time * delay + trial_number + after_nth_view_s
es +      (1 + cumul_view_time * delay | id)
   Data: df

     AIC      BIC   logLik deviance df.resid 
 17898.6  18022.5  -8933.3  17866.6    16954 

Scaled residuals: 
    Min      1Q  Median      3Q     Max 
-19.147  -0.716   0.416   0.635 240.610 

Random effects:
 Groups Name                  Variance Std.Dev. Corr             
 id     (Intercept)           0.363128 0.60260                   
        cumul_view_time       0.156921 0.39613  -0.15            
        delay                 0.019284 0.13887  -0.45 -0.25      
        cumul_view_time:delay 0.002811 0.05302   0.14  0.12 -0.85
Number of obs: 16970, groups:  id, 46

Fixed effects:
                       Estimate Std. Error z value Pr(>|z|)    
(Intercept)            0.734725   0.128745   5.707 1.15e-08 ***
cumul_view_time        0.478942   0.094434   5.072 3.94e-07 ***
delay                 -0.297404   0.024739 -12.022  < 2e-16 ***
trial_number           0.034513   0.002224  15.517  < 2e-16 ***
after_nth_view_ses     0.070997   0.009405   7.549 4.39e-14 ***
cumul_view_time:delay  0.059991   0.015374   3.902 9.54e-05 ***
---
Signif. codes:  0 ‘***’ 0.001 ‘**’ 0.01 ‘*’ 0.05 ‘.’ 0.1 ‘ ’ 1

Correlation of Fixed Effects:
            (Intr) cml_v_ delay  trl_nm aft___
cumul_vw_tm -0.472                            
delay       -0.548  0.175                     
trial_numbr -0.294  0.042 -0.008              
aftr_nth_v_ -0.294 -0.001  0.018  0.160       
cml_vw_tm:d  0.420 -0.521 -0.756 -0.020 -0.010
optimizer (Nelder_Mead) convergence code: 0 (OK)
Model failed to converge with max|grad| = 0.0269237 (tol = 0.002, component
1)
```
