# Supplementary material for: The rise and fall of memories: Temporal dynamics of visual working memory
Source: Mem Cognit. 2025 May 6;53(8):2406–23. doi: 10.3758/s13421-025-01718-9 (PMC12695987; doi:10.3758/s13421-025-01718-9)

Supplementary material B

Below we report the performance in Experiment 2A in terms of absolute error, across the 33 repetitions of the nine unique trials they encountered throughout the experiment. We spilt the data for set size one and three, as we manipulated set size *between* participants.

In the set size 3 condition there seems to be no improvement at all. In the set size 1 condition participants seem to get better as the absolute error decreases. But some improvement is to be expected, considering participants get better at any task merely due to learning effects. It is perhaps a bit more surprising that there is no apparent improvement in the set size 3 condition.

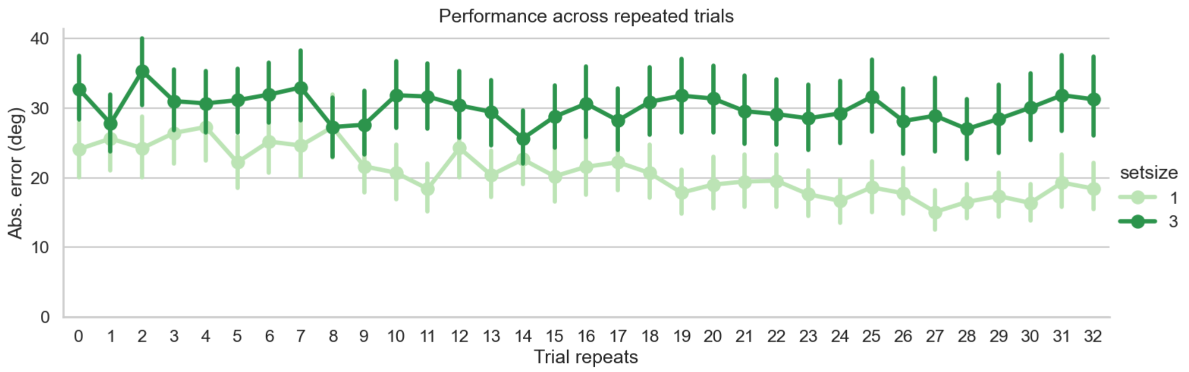

Supplement: Supplementary file 2 — (pdf 211 KB) [file 13421_2025_1718_MOESM2_ESM.pdf]
