# Supplementary figures and images for: The rise and fall of memories: Temporal dynamics of visual working memory
Source: Mem Cognit. 2025 May 6;53(8):2406–23. doi: 10.3758/s13421-025-01718-9 (PMC12695987; doi:10.3758/s13421-025-01718-9)

## Experiment 2A

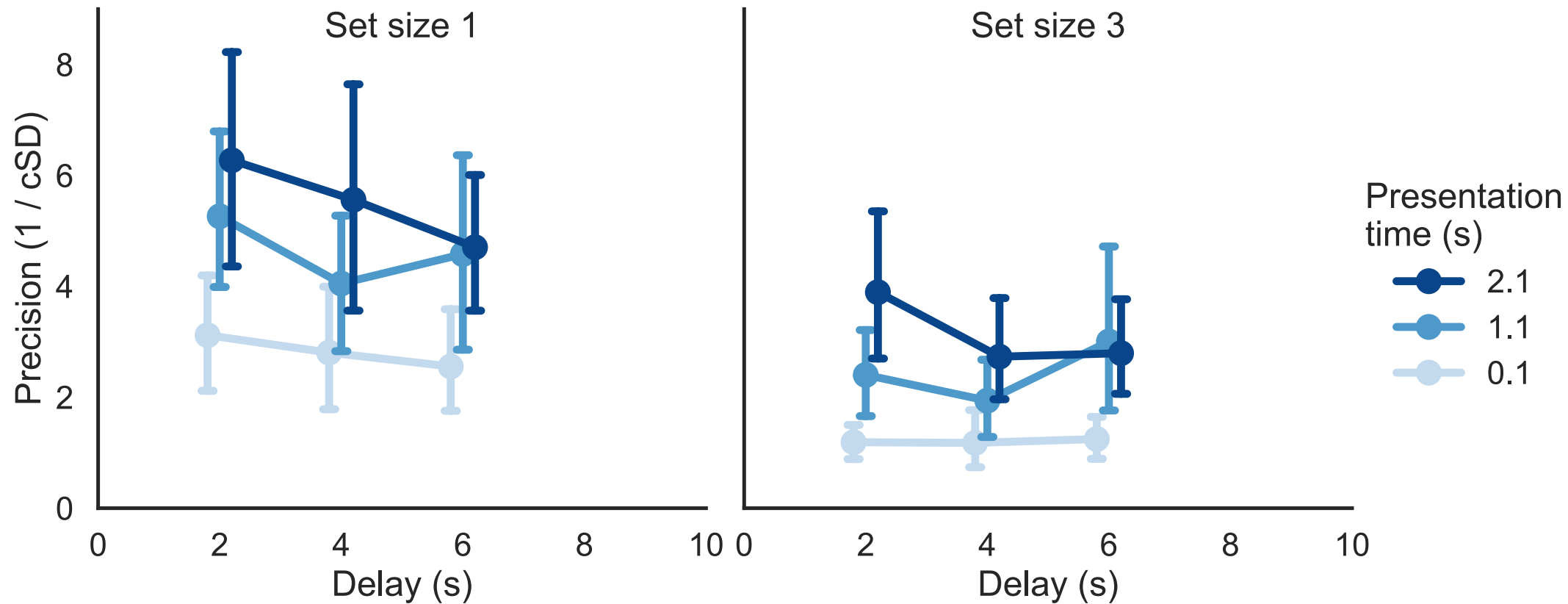

Supplement: Supplementary file 3 — (pdf 46 KB) [file 13421_2025_1718_MOESM3_ESM.pdf]
